# Senkyunolide I suppresses hepatic stellate cell activation and liver fibrosis by reprogramming VDR-dependent fatty acid metabolism

**DOI:** 10.1186/s13020-025-01133-x

**Published:** 2025-06-13

**Authors:** Mengyao Zhu, Lu Ren, Wenlong Xiao, Longjian Wang, Zhiming Hu, Nani Wang

**Affiliations:** 1 School of Pharmacy, Hangzhou Medical College, Hangzhou, 310007 Zhejiang China; 2 Tongde Hospital of Zhejiang Province, Zhejiang Chinese Medical University, Hangzhou, 310007 Zhejiang China; 3 Department of Medicine, Zhejiang Academy of Traditional Chinese Medicine, Hangzhou, 310007 Zhejiang China

**Keywords:** Senkyunolide I, Fatty acid metabolism reprogram, Vitamin D receptor, Liver fibrosis, Hepatic stellate cells

## Abstract

Hepatic stellate cells (HSCs) activation represents a central pathological mechanism in liver fibrosis, with emerging evidence implicating fatty acid metabolic reprogramming as a critical regulator of this process. Our study established the vitamin D receptor (VDR) as a key transcriptional coordinator of fatty acid metabolism during HSC activation. Genetic VDR deletion in mice exacerbated liver fibrosis progression, which was associated with elevated TGF-β1 levels and increased Smad3 phosphorylation. Mechanistically, VDR deficiency disrupted lipid homeostasis through the upregulation of lipogenic enzymes (fatty acid synthase, acetyl-CoA carboxylase 1, ATP citrate lyase) and desaturases (stearoyl-CoA desaturase-1, fatty acid desaturases 1/2) and the suppression of the β-oxidation gatekeeper carnitine palmitoyltransferase 1A (CPT1A). Pathological VDR downregulation was observed in both TGF-β1-activated HSCs and fibrotic liver tissues, suggesting a disease-associated regulatory circuit. Calcitriol-mediated VDR activation reversed TGF-β1-induced Smad3 phosphorylation and normalized metabolic enzyme expression, effectively reducing lipid accumulation and collagen deposition. We further identified senkyunolide I as a novel natural VDR agonist that rebalances fatty acid metabolism by simultaneously downregulating lipogenesis/desaturation machinery and upregulating CPT1A. The complete abolition of anti-fibrotic effects of senkyunolide I following VDR knockdown confirmed its strict receptor dependency. These findings identify VDR as a master regulator of metabolic reprogramming in HSC activation and validate pharmacological VDR activation as a promising therapeutic strategy for liver fibrosis. The dual metabolic regulatory capacity of senkyunolide I through VDR signaling highlights its potential for targeted antifibrotic intervention.

**Supplementary Information:**

The online version contains supplementary material available at 10.1186/s13020-025-01133-x.

## Introduction

Liver fibrosis represents a pivotal pathological process in chronic liver disease progression [44] and is characterized by excessive deposition and aberrant remodeling of extracellular matrix (ECM) components within hepatic tissue [40]. This condition has grave clinical implications, with epidemiological studies showing a median survival of 2–3 years postdiagnosis and five-year survival rates of < 30%. Current antiviral, including therapies-including entecavir, adefovir, and telbivudine, demonstrate clinical efficacy in viral suppression [28], although their utility is limited by dose-limiting toxicity. These adverse effects include gastrointestinal reactions, hepatic damage, visual impairment, and renal dysfunction, which collectively compromise therapeutic sustainability and negatively impact patient prognosis [46].

The activation of hepatic stellate cells (HSCs) represents a pivotal mechanism in liver fibrogenesis [22], and is characterized primarily by their differentiation into myofibroblasts and excessive extracellular matrix (ECM) deposition. Emerging evidence highlights that dysregulated fatty acid metabolism in activated HSCs exacerbates hepatic lipid accumulation and fibrosis progression, creating a self-perpetuating pathogenic cycle [41, 47]. At the molecular level, transforming growth factor-β1 (TGF-β1) orchestrates HSCs activation by inducing α-smooth muscle actin (α-SMA) expression and fibrogenic phenotype [21]. Besides, TGF-β1 upregulates key lipogenic enzymes including fatty acid synthase (FASN), acetyl-CoA carboxylase 1 (ACC1), ATP-citrate lyase (ACLY), fatty acid desaturase (FADS1/2), and stearoyl-CoA desaturase 1 (SCD1) [13], while simultaneously suppressing carnitine palmitoyltransferase 1 A (CPT1 A), the rate-limiting enzyme in fatty acid oxidation [51]. This dual modulation shifts HSCs toward lipid anabolism and impairs β-oxidation, resulting in intracellular lipid overload. The Smad3 signaling pathway serves as a critical mediator of these effects, further reinforcing the profibrotic microenvironment through disrupted hepatic lipid homeostasis and amplified ECM deposition [27]. Notably, pharmacological inhibition of these dysregulated metabolic nodes (particularly FASN, ACC1, and SCD1) has demonstrated efficacy in attenuating HSCs activation and reducing collagen accumulation [34], positioning fatty acid metabolism as a promising therapeutic target for preventing fibrosis progression.

The vitamin D receptor (VDR) plays a pivotal role in regulating lipid homeostasis in HSCs. Ligand-activated VDR has been shown to effectively suppress HSCs activation and attenuate extracellular matrix deposition in hepatic fibrosis [48]. Experimental studies using obese mouse models demonstrate that VDR activation reduces fatty acid and cholesterol levels [42], whereas VDR deficiency aggravates diet-induced hepatic lipid accumulation and promotes chronic inflammation [12, 23]. Mechanistic investigations reveal that VDR agonists modulate key fatty acid metabolism enzymes such as ACC-1 and FASN [48], with effects observed in multiple tissue types including adipose tissue, adipose-derived mesenchymal stem cells [35], and colorectal tumorigenesis models [52]. However, the exact molecular mechanisms through which VDR maintains lipogenesis balance during HSCs activation remain to be fully elucidated.

In this study, we demonstrated that VDR deficiency exacerbates hepatic fibrosis by triggering metabolic reprogramming in HSCs. Mechanistically, VDR depletion activates the TGFβ1/Smad3 signaling pathway, leading to the transcriptional upregulation of lipogenic enzymes (FASN, ACC-1, ACLY) and fatty acid desaturases (SCD1, FADS1/2), while simultaneously suppressing CPT1 A. This metabolic dysregulation results in lipid accumulation and enhanced ECM deposition in activated HSCs. Importantly, we discovered senkyunolide I (SI) as a novel VDR agonist that specifically targets the ligand-binding domain. SI demonstrates potent anti-fibrotic effects in CCl_4_-induced fibrotic mice by normalizing lipid metabolism homeostasis and inhibiting TGF-β1/Smad3 signaling, without causing hypercalcemia. These findings establish VDR-mediated metabolic regulation as a promising therapeutic target and underscore the SI's clinical potential for treating liver fibrosis.

## Materials and methods

### Animal treatment

Male C57BL/6 mice (8-weeks-old) were obtained from Hangzhou Medical College (Zhejiang, China) and housed under specific pathogen-free conditions with a 12-h light/dark cycle, ad libitum access to food and water. VDR-knockout (VDR^−/−^) and wild-type (WT) mice on a C57BL/6 background were generated as previously described [30]. Hepatic fibrosis was induced via intraperitoneal (i.p.) injection of 10% CCl_4_ (C112045, Aladdin, Shanghai, China; i.p.) three times a week [3]. The vehicle control group received equivalent volumes of olive oil (O108686, Aladdin). CCl_4_-treated mice were randomized into five groups: model group, low-dose SI (SI-L, 50 mg/kg, i.p.; YJ0118, Yongjian Pharmaceutical, Jiangsu, China), high-dose SI (SI–H, 100 mg/kg, i.p.) [49], positive drug silybin (SLB, 20 mg/kg, i.p.,HY-N0779 A, Yongjian Pharmaceutical) [37], and positive drug calcitriol (VD, 5 µg/kg, i.p.,#C120126, Aladdin) [38].

### Assessment of liver function parameters

The alanine aminotransferase (ALT), aspartate aminotransferase (AST) alkaline phosphatase (ALP) activity, and total bilirubin (TBIL) concentrations were quantified using commercial kits (C009-2-1, C010-2-1, A059-2-2, and C019-1-1; Nanjing Jiancheng Bioengineering Institute, Jiangsu, China). The absorbance was recorded using a SpectraMax 190 microplate reader (Molecular Devices, USA).

### Histopathological analysis

Right hepatic lobes were fixed in 10% neutral buffered formalin and paraffin-embedded. Hematoxylin–eosin (HE) staining was performed using a standardized protocol [25] with a commercial kit (G1076, Servicebio, Hubei, China). Histological sections were examined under an APERIO VERSA 8 microscope (Leica, Germany).

### Collagen deposition assessment

Sirius red staining was conducted following established protocols [11] using a specialized staining kit (HK2021, Servicebio). Collagen fiber distribution was visualized and documented microscopically.

### Fibrosis Evaluation

Masson's trichrome staining was performed according to validated methods [5] with a commercial kit (G1006, Servicebio), enabling differential visualization of collagen fibers and cellular components.

### Immunohistochemistry

Liver sections were immunostained using validated protocols [53, 55] with the following primary antibodies: anti-TGF-β1 (BF8012, Affinity, Jiangsu, China,1:200), anti-p-Smad3 (AF8315, Affinity; 1:200), anti-α-SMA (BF9212, Affinity; 1:200), and anti-collagen type I α 1 (COL1 A1, PB0981, Boster; Hubei; 1:200) and imaged under a confocal laser scanning microscope (LSM800, Zeiss, Oberkochen, Germany).

### Cell culture and treatment

HSCs were obtained from the Chinese Academy of Sciences Type Culture Collection and maintained in DMEM supplemented with 2% FBS (11,995,065, Gibco) at 37 °C with 5% CO_2_. Cells were divided into nine experimental groups: (1) untreated control; (2) TGF-β1 model (10 ng/mL, Ag24881, Proteintech, Hubei, China) [8], (3–5) TGF-β1 + SI (20, 50, 100 μM) [43], (6–7) TGF-β1 + VD (8, 16 μM) [36]; (8) TGF-β1 + SLB (100 μM) [17]; (9) TGF-β1 + TVB3664 (TVB, 150 nM, 2097262-58-1, MedChemExpress, NJ, USA) [32]. After 24 h treatment, cells were harvested for downstream analysis.

### Cell viability

Cell viability was determined via 3-(4,5-dimethylthiazol-2-yl)−2,5-diphenyltetrazolium bromide kit (C0009S, Beyotime) with absorbance measurements at 490 nm (Spectra MAX 190, Molecular Devices). Results were normalized to untreated controls.

### Quantitative RT-PCR analysis

Total RNA was isolated using TRIzol (99,089,501, Invitrogen, CA, US) and reverse transcribed with SweScript RT SuperMix (G3337, Servicebio). Gene expression of *α-Sma*, *Col1a1*, *Timp1*, *Cpt1a*, *Fasn*, *Fads1*, *Fads2, Acc-1*, *Acly*, and *Scd1* was quantified on a 7500 Real-Time PCR System (Applied Biosystems, USA) using SYBR Green Master Mix (G3326, Servicebio). GAPDH served as endogenous control, with primer sequences detailed in Table S1.

### Western blot

Total protein extracts were prepared using KeyGen Kits (KGP250, KeyGen), with nuclear fractions isolated via a nuclear and cytoplasmic protein extraction kit (P0027, Beyotime). After Bradford quantification (KGA801, KeyGen), proteins were separated by SDS-PAGE and transferred to PVDF membranes. Membranes were blocked with 5% BSA and probed overnight at 4 °C with antibodies against: α-SMA (1:2000), COL1 A1 (1:1000), FASN (10624-2-AP, Proteintech, 1:1000), VDR (AF8159, Affinity, 1:1000), p-Smad3 (1:2000), Smad3 (AF6362, Affinity, 1:2000), TGF-β1 (1:3000) and β-actin (BM3873, Boster, 1:2000). HRP-conjugated secondary antibodies were used for chemiluminescent detection (ChemiDoc MP, Bio-Rad, CA, USA).

### RNA interfection

HSCs were transfected with VDR-targeting siRNA (si-VDR, GenePharma, Shanghai, China) and negative control siRNA (si-NC) using Lipo6000 transfection reagent (C0526, Beyotime). The sequences of the siRNAs used are provided in Table S2. The knockdown efficiency was confirmed through western blot analysis of VDR protein expression. The experimental groups included the following: 1) si-NC; 2) si-VDR; 3) si-NC + TGF-β1 (10 ng/mL); 4) si-VDR + TGF-β1; 5) si-NC + TGF-β1 + SI (100 μM); and 6) si-VDR + TGF-β1 + SI. The cells were harvested 24 h post-treatment for subsequent analyses.

### Immunofluorescence staining

Fixed cells (4% paraformaldehyde) were permeabilized with 0.1% Triton X-100, and then incubated overnight at 4 °C with the following primary antibodies: anti-α-SMA and anti-CT-8 (PB2553, Proteintech; 1:500). Alexa Fluor-conjugated secondary antibodies were applied for 1 h. Nuclei were counterstained with DAPI (C1005, Beyotime). Images were acquired using a Zeiss LSM510 META confocal microscope and analyzed with ZEN imaging software.

### Neutral lipid quantification

The cells were fixed and stained with Nile Red (C2051S, Beyotime) to detect cytoplasmic neutral lipid accumulation. Fluorescence intensity was quantified using Zeiss LSM510 META with 543 nm excitation/598 nm emission filters.

### Molecular docking

The 3D structure of theVDR (PDB ID: 1 KB2) was retrieved from RCSB Protein Data Bank. The 2D structure of SI (PubChem CID: 3,085,257) was converted to 3D using Chem3D [31]. Molecular docking was performed in AutoDock 4.2. The results were visualized using PyMOL [4].

### Cellular thermal shift assay (CETSA)

Protein aliquots were thermally challenged at 30, 40, 50, and 60 °C for 3 min to induce temperature-dependent denaturation [10]. Following incubation, samples were mixed with loading buffer and analyzed by western blot to determine the thermal stability profile of VDR.

### Statistical analysis

Statistical analyses were performed using GraphPad Prism 8 (GraphPad Software, CA, USA). Normality and homogeneity of variances were confirmed before implementing Student's t-test for two-group comparisons and one-way ANOVA for multigroup analyses. Following significant ANOVA results (*P* < 0.05), Tukey's honestly significant difference test was employed for post hoc pairwise comparisons. Continuous data are expressed as mean ± standard deviation (SD), with P values < 0.05 considered statistically significant.

## Results

### VDR ablation exacerbates liver fibrosis and disrupts fatty-acid metabolism in mice

**Fig. 1:**
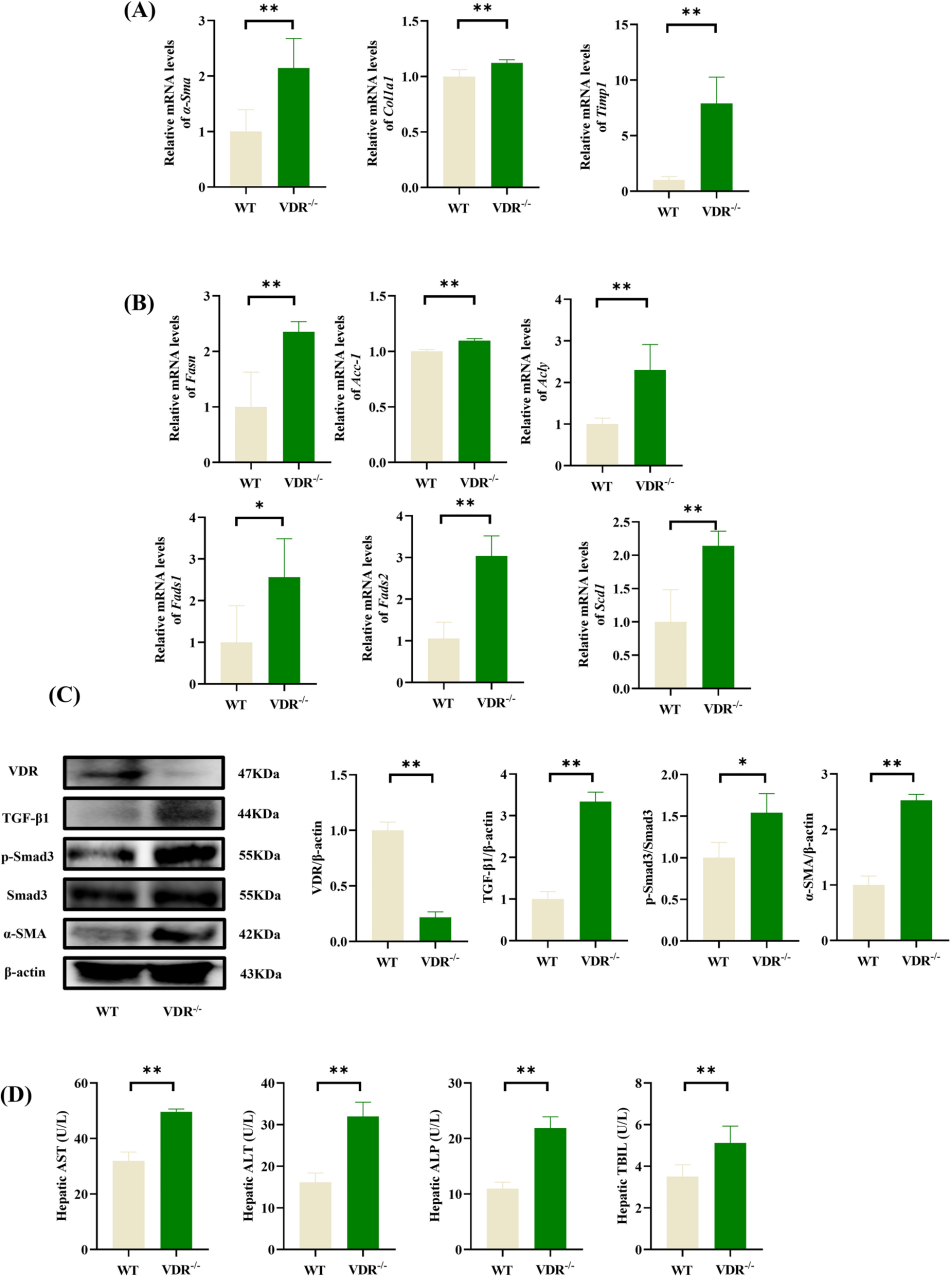
VDR deficiency exacerbated hepatic fibrosis and steatosis in mice. **A** mRNA levels of fibrogenic markers (*α-Sma*, *Col1a1*, and *Timp1*). **B** mRNA levels of lipogenic enzymes (*Fasn*, *Acc-1*, and *Acly*) and fatty acid desaturases (*Fads1/2* and *Scd1*) (n = 5). **C** Western blot analysis of VDR and TGFβ/Smad pathway (n = 3), **D** Serum levels of liver function markers (AST, ALT, ALP, and TBIL) (n = 5). *P < 0.05, **P < 0.01

VDR, a nuclear receptor, exerts protective effects against inflammation and fibrogenesis by modulating transcriptional pathways [9]. To investigate the role of VDR in liver pathology, we analyzed the expression of profibrotic markers (*α-Sma*, *Col1a1*, and *Timp1*) [2, 22] and fatty acid metabolism regulators in VDR-deficient mice. Compared with those in WT controls, hepatic mRNA expression of *α-Sma*, *Col1a1*, and *Timp1* was significantly elevated in VDR^−/−^ mice (Fig. 1A), indicating aggravated fibrotic progression. Concurrently, VDR ablation markedly upregulated lipogenic enzymes (*Fasn*, *Fads1*, and *Fads2*) and key fatty acid synthesis regulators (*Acc-1*, *Acly*, and *Scd1*) (Fig. 1B), suggesting profound dysregulation of lipid homeostasis.

Western blot analysis revealed that VDR knockout mice exhibited elevated hepatic protein levels of TGF-β1 and α-SMA (Fig. 1C), which was consistent with enhanced fibrotic signaling. Phosphorylated Smad3 (p-Smad3), a downstream effector of TGF-β1, was significantly increased in the livers of VDR^−/−^ livers. The levels of serum ALT, AST, ALP and TBIL levels, biomarkers of hepatocellular injury, were also elevated in VDR^−/−^ mice (Fig. 1D), further supporting the role of VDR in mitigating liver damage.

### VDR activation mitigates TGF-β1-induced metabolic dysregulation and fibrogenesis in HSCs

**Fig. 2:**
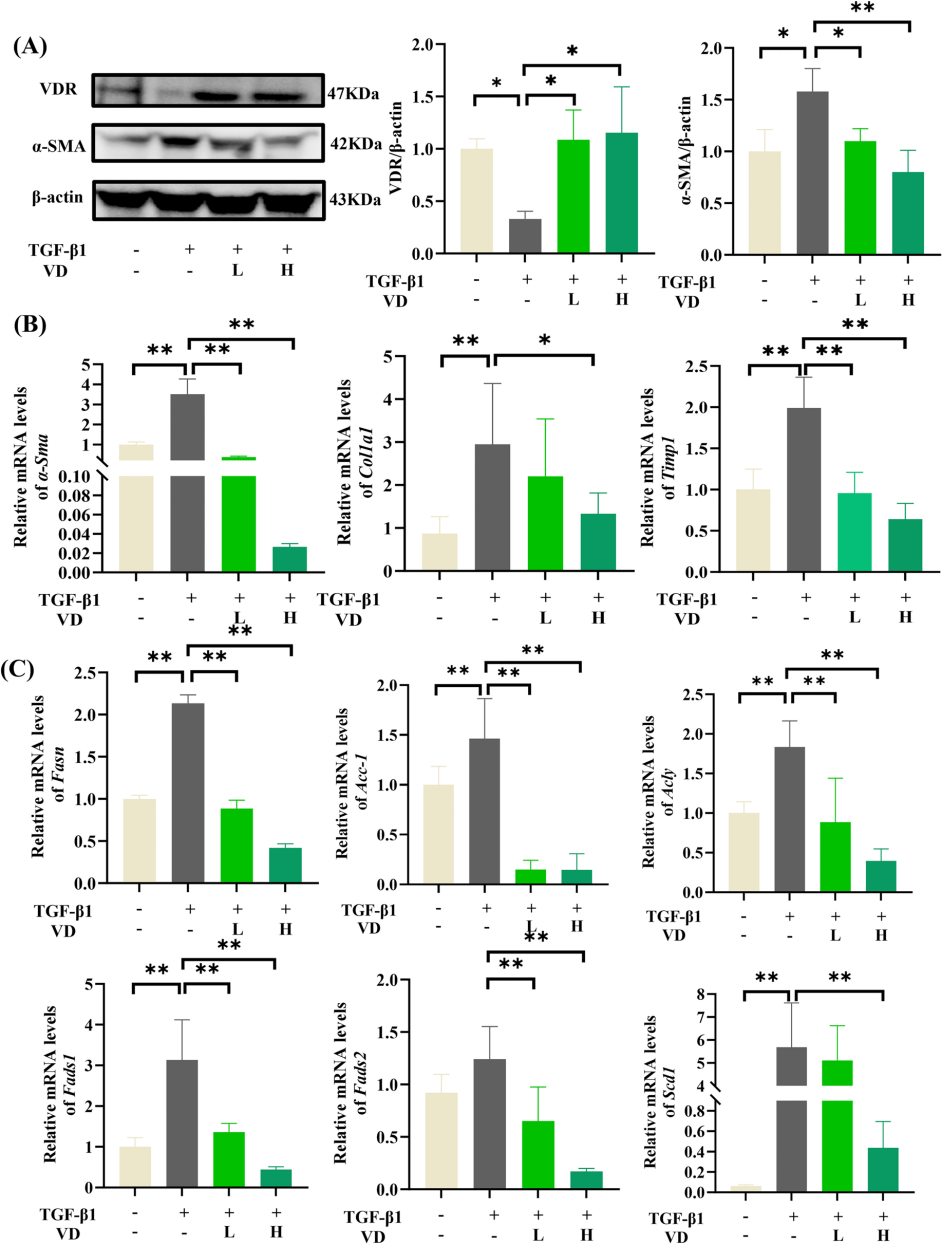
VDR activation attenuated TGF-β1-induced fibrosteatotic changes in HSCs. **A** Western blot analysis of VDR and α-SMA (n = 3). **B** mRNA levels of fibrogenic markers (*α-Sma*, *Col1a1*, and *Timp1*). **C** mRNA levels of lipogenic enzymes (*Fasn*, *Acc-1*, and *Acly*) and fatty acid desaturases (*Fads1/2* and *Scd1*) (n = 6). *P < 0.05, **P < 0.01

TGF-β1 stimulation significantly suppressed VDR expression (Fig. 2A) while elevating key fibrotic markers *α-Sma*, *Col1a1*, and *Timp1* in HSCs (Fig. 2B). Calcitriol treatment dose-dependently reversed the TGF-β1-induced downregulation of VDR expression and upregulation of fibrotic markers.

To investigate the metabolic regulatory mechanism, we analyzed mRNA expression of lipogenic enzymes, which showed significant elevation in TGF-β1-stimulated HSCs compared to controls (Fig. 2C). Calcitriol administration normalized these metabolic perturbations, demonstrating that VDR activation exerts dual anti-fibrotic and lipid-homeostatic effects in activated HSCs.

### *SI attenuates TGF-β1-mediated HSCs activation *via* VDR interaction*

**Fig. 3:**
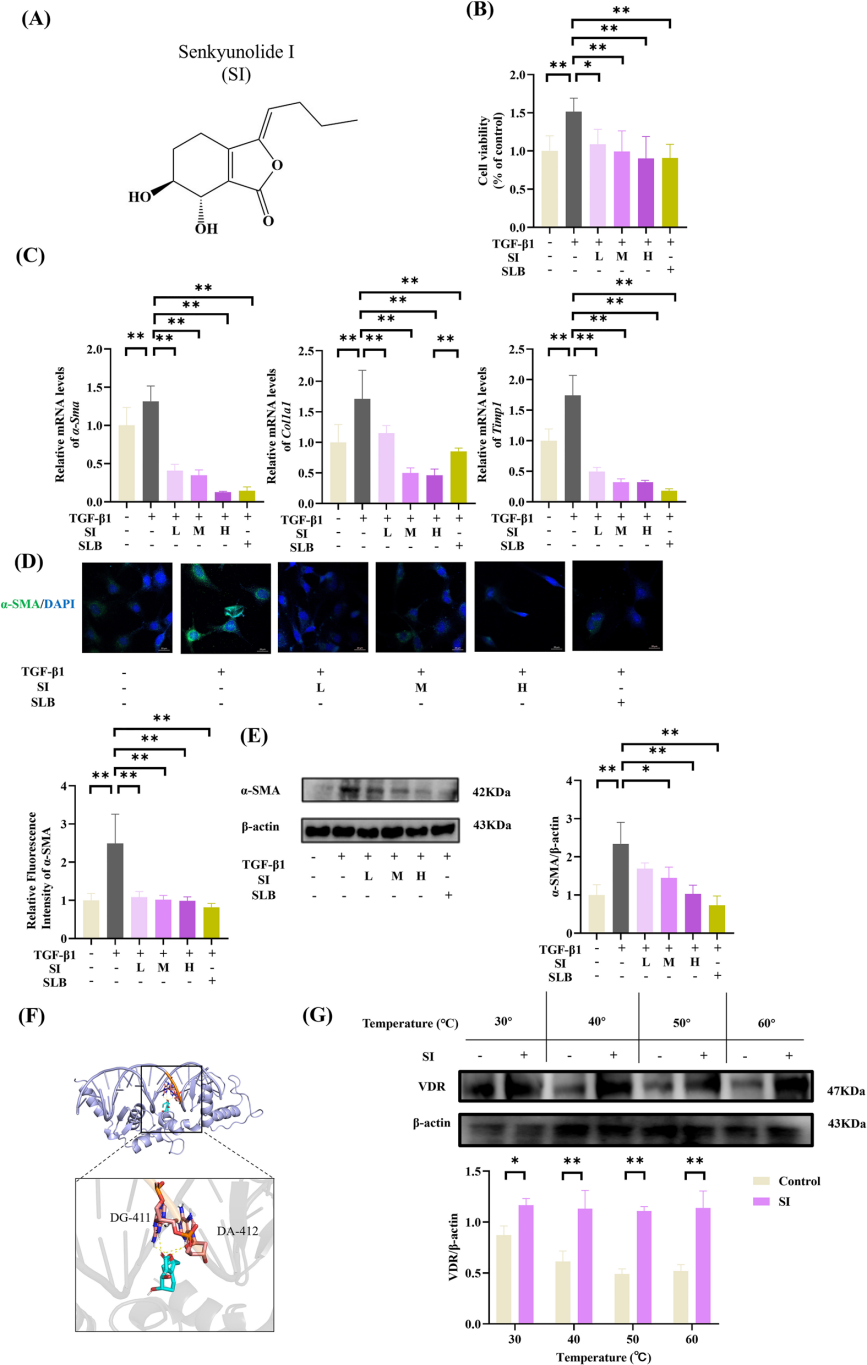
SI inhibited TGF-β1-induced activation of HSCs. **A** Chemical structure of the SI. **B** Cell viability of HSCs. **C** mRNA levels of fibrogenic markers (*α-Sma*, *Col1a1*, and *Timp1*). **D** Immunofluorescence analysis of α-SMA. scale bar = 20 μm. (n = 6). **E** Western blot analysis of α-SMA. **F** Molecular docking results of VDR and SI. **G** CETSA analysis. (n = 3) *P < 0.05, **P < 0.01

We investigated the inhibitory effects of SI on TGF-β1-induced HSCs activation. As shown in Fig. 3A, B, SI significantly reversed the TGF-β1 induced increase in HSCs. Compared with those of the controls, RT-PCR revealed dose-dependent suppression of fibrotic markers (*α-Sma*, *Col1a1*, and *Timp1*) by SI in the TGF-β1-stimulated cells (Fig. 3C). Immunofluorescence (Fig. 3D) and western blot (Fig. 3E) analyses confirmed SI-mediated reduction of α-SMA protein expression in TGF-β1-activated HSCs.

Molecular docking simulations predicted stable binding between SI and VDR with low binding energy (− 7.0 kcal/mol), suggesting direct interaction (Fig. 3F). CETSA demonstrated the ability of SI to stabilize VDR against thermal degradation at 30–60 °C (Fig. 3G).

### *SI ameliorates CCl*_*4*_*-induced liver fibrosis in mice*

**Fig. 4:**
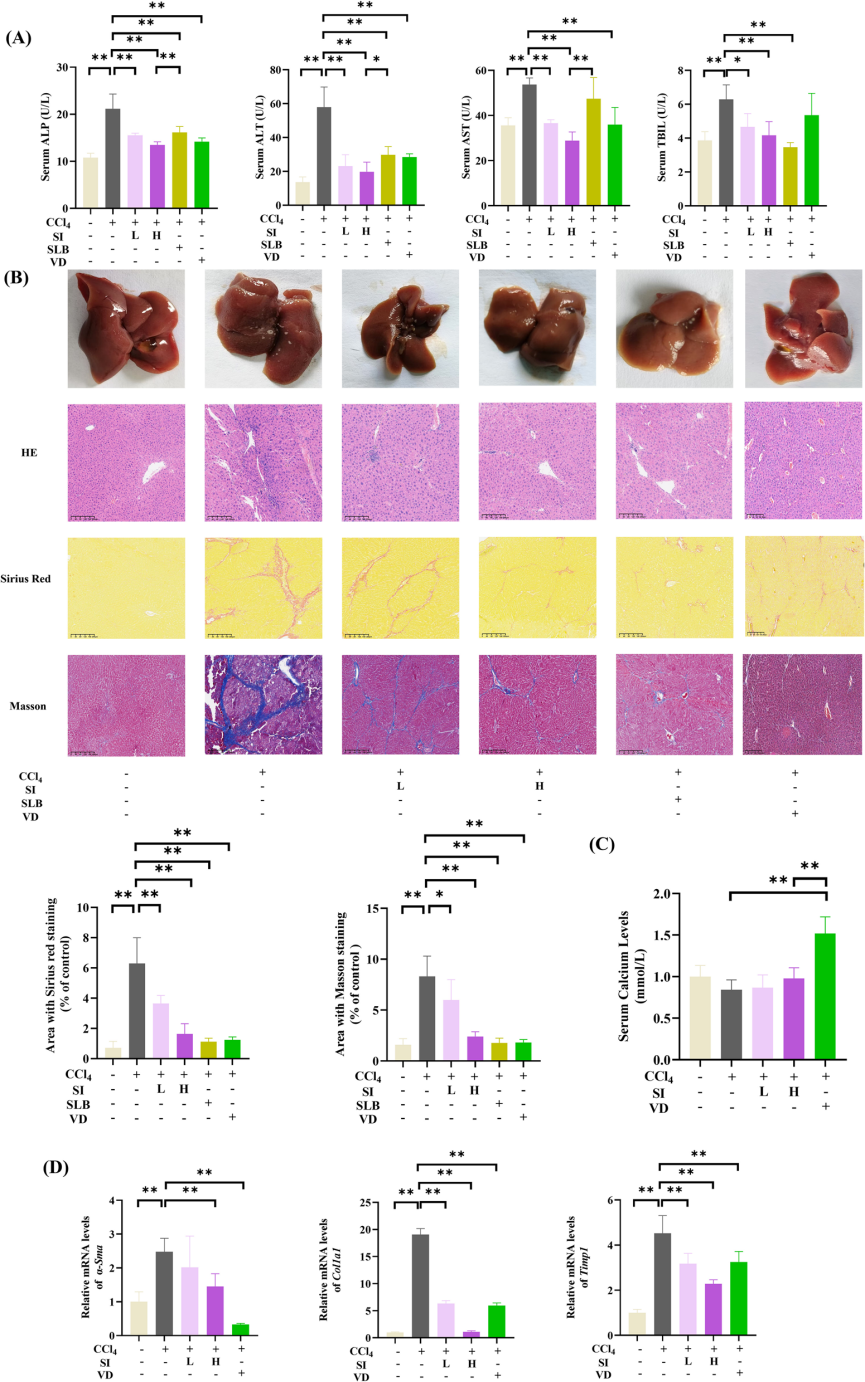
SI ameliorated CCl_4_-induced liver fibrosis in mice. **A** Serum levels of liver function markers (AST, ALT, ALP, and TBIL). **B** Liver tissue photograph and histopathological analysis (HE, Sirius Red, Masson), scale bar = 200 μm. **C** Serum calcium concentrations. **D** mRNA levels of fibrogenic markers (*α-Sma*, *Col1a1*, and *Timp1*). (n = 5) *P < 0.05, **P < 0.01

To assess the therapeutic efficacy of SI against hepatic fibrosis, we established a CCl_4_-induced murine liver fibrosis model. SI and VD treatment attenuated CCl_4_-driven elevations in the levels of serum markers of hepatic injury, including ALP, ALT, AST, and TBIL (Fig. 4A), demonstrating its hepatoprotective effects. Additionally, our study demonstrated no observable toxicity associated with SI administration in the liver fibrosis model. All animals treated with SI exhibited a 100% survival rate.

Gross morphological analysis revealed that CCl_4_-exposed livers presented rough surfaces with coarse granularity, blunt edges, and hardened texture. The interventions of SI, silybin, and VD improved hepatic architecture, reducing surface irregularities and fibrosis severity (Fig. 4B).

Histopathological evaluation by HE staining confirmed extensive hepatocyte disarray, inflammatory infiltration, and focal necrosis in the CCl_4_ group. The treatments of SI, silybin, and VD markedly reduced necrotic areas and inflammatory cell recruitment. We found that CCl_4_ did not cause fibrosis in kidney, and SI had no significant effect on renal α-SMA and COL1 A1 expression levels (Fig. S1).

Masson’s trichrome staining, a validated method for fibrosis quantification [54], further demonstrated that CCl_4_ exposure disrupted hepatic lobular architecture, inducing marked fibrous septa formation surrounding parenchymal structures (pseudolobulation). SI administration significantly decreased collagen deposition compared with that in the model group.

Notably, SI did not induce hypercalcemia, as serum calcium levels remained unchanged compared with those in the model group (Fig. 4C). In contrast, VD treatment caused significant hypercalcemia, which is consistent with the known calcium-regulatory properties of VD. RT-PCR analysis demonstrated marked upregulations of *α-Sma*, *Timp1* and *Col1a1* mRNA levels in the model group compared with those in the control group (Fig. 4D), which was attenuated by SI in a dose-dependently manner. These results collectively indicate that SI alleviates CCl_4_-induced hepatic fibrosis without perturbing calcium homeostasis.

### SI modulated fatty acid metabolism and ameliorated TGF-β/Smad3 pathway in liver.

**Fig. 5:**
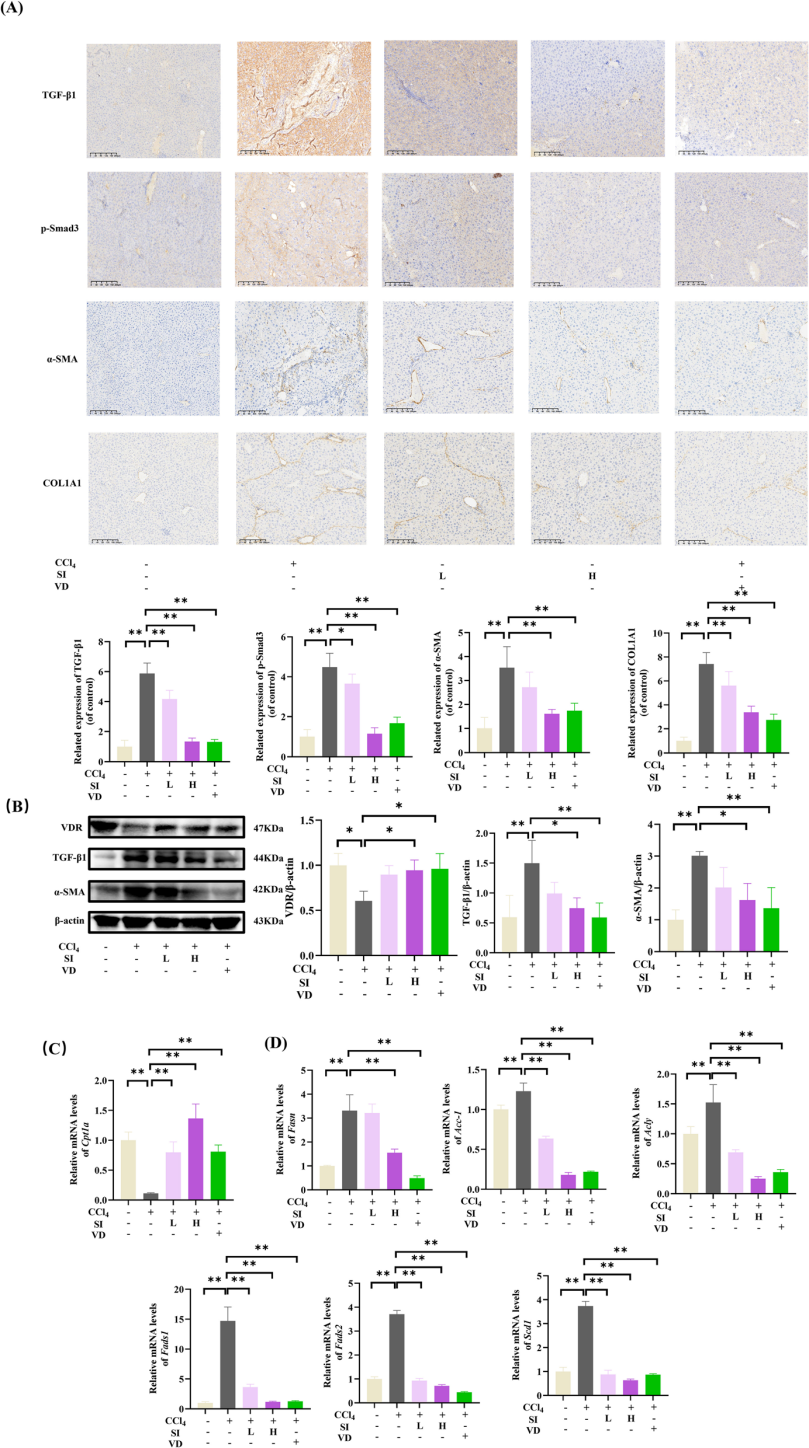
SI regulated VDR and fatty acid metabolism to suppress TGFβ/Smad pathway in CCl_4_-induced mice. **A** Immunohistochemistry analysis of TGF-β1, p-Smad3, α-SMA, COL1 A1. scale bar = 200 μm. (n = 5). **B** Western blot analysis of VDR, TGF-β1, and α-SMA. **C** mRNA levels of *Cpt1a*. **D** mRNA levels of lipogenic enzymes (*Fasn*, *Acc-1*, and *Acly*) and fatty acid desaturases (*Fads1/2* and *Scd1*) (n = 6) *P < 0.05, **P < 0.01

Immunohistochemical analysis revealed that CCl_4_-induced elevations in hepatic TGF-β1, p-Smad3, α-SMA, and COL1 A1 protein levels were similarly suppressed by SI treatment in a dose-dependent manner (Fig. 5A). Western blot analysis confirmed the ability of SI to reverse CCl_4_-induced α-SMA overexpression (Fig. 5B). Notably, compared with control treatment, CCl_4_ administration significantly downregulated VDR expression, whereas SI and VD treatment restored VDR activation. This VDR activation correlated with SI-mediated metabolic reprogramming, as evidenced by reversal of CCl_4_-induced alterations in lipid metabolism enzymes: decreased *Cpt1a* (Fig. 5C) and increased *Fasn*, *Fads1*, *Fads2*, *Acc-1*, *Acly* and *Scd1* levels (Fig. 5D) were normalized following SI intervention.

### SI regulated lipid metabolism reprogramming in TGF-β1-induced HSCs

**Fig. 6:**
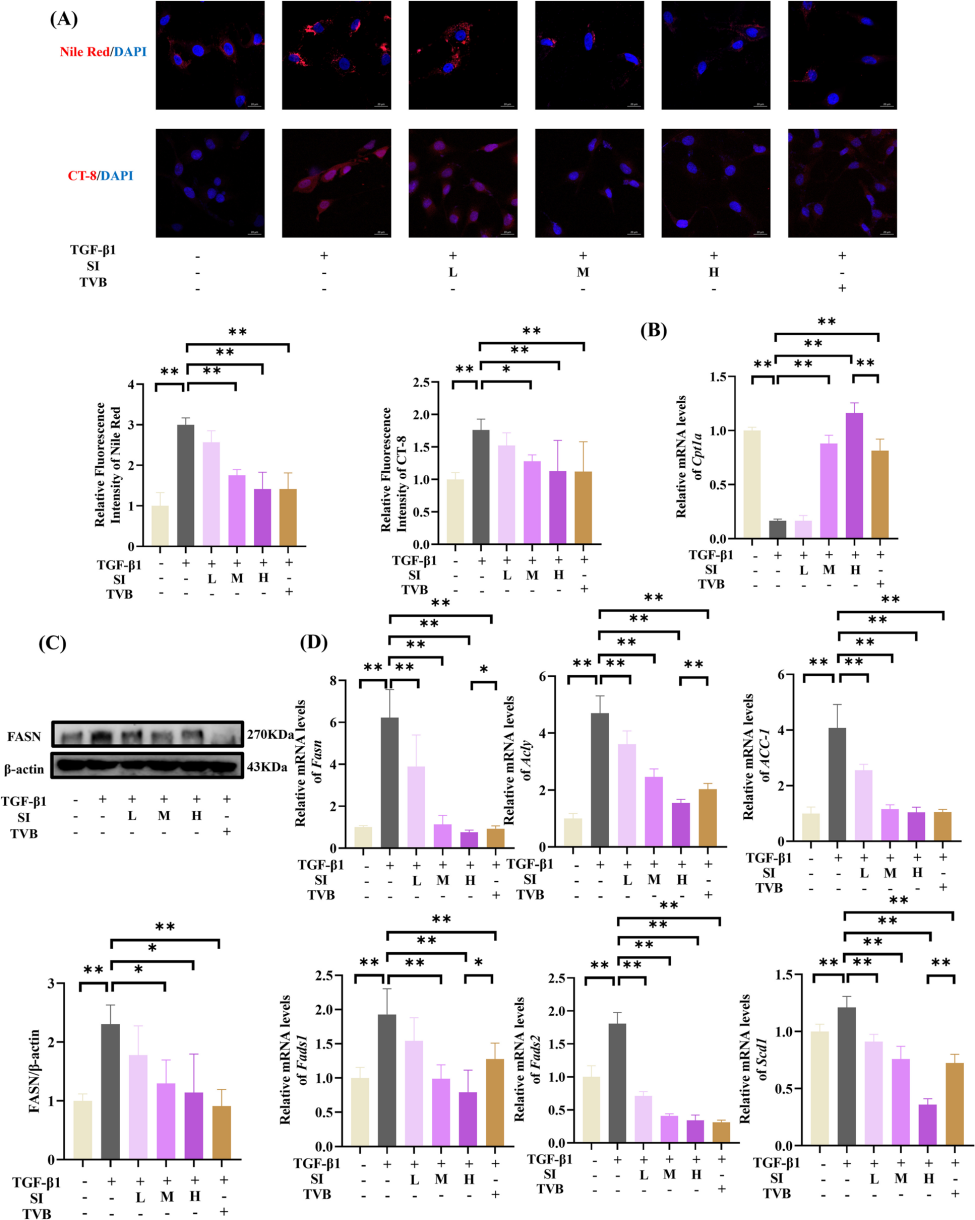
SI inhibited lipid accumulation through regulating fatty acid metabolism in TGF-β1-induced HSCs. **A** lipid droplet accumulation assessed by Nile Red and CT-8. scale bar = 20 μm. (n = 6). **B** *Cpt1a* expression. **C** Western blot analysis of FASN. (n = 3). **D** mRNA levels of lipogenic enzymes (*Fasn*, *Acc-1*, and *Acly*) and fatty acid desaturases (*Fads1/2* and *Scd1*) (n = 6) *P < 0.05, **P < 0.01

Aberrant fatty acid metabolism contributes to hepatic lipid accumulation and fibrosis progression. We investigated SI's regulatory effects on lipid metabolism in TGF-β1-activated HSCs. Nile Red staining^18^ revealed that TGF-β1 exposure induced intracellular lipid accumulation (Fig. 6A), which was attenuated by SI treatment. Compared with that of their TGF-β1-activated counterparts, the fluorescence intensity of SI-treated HSCs was significantly lower via complementary CT-8 quantification, confirming the capacity of SI to mitigate lipid droplet formation.

Mechanistically, TGF-β1 activation downregulated *Cpt1a* (Fig. 6B), while upregulating FASN protein levels (Fig. 6C). SI treatment restored CPT1 A expression and normalized FASN overexpression. Transcriptomic analysis further showed TGF-β1-induced upregulation of lipogenic genes including *Fads1*, *Fads2, Acc-1*, *Acly*, and *Scd1* (Fig. 6D), which were significantly suppressed by both SI and the reference inhibitor TVB.

### SI attenuates TGF-β1-induced HSCs activation through VDR pathway activation

**Fig. 7:**
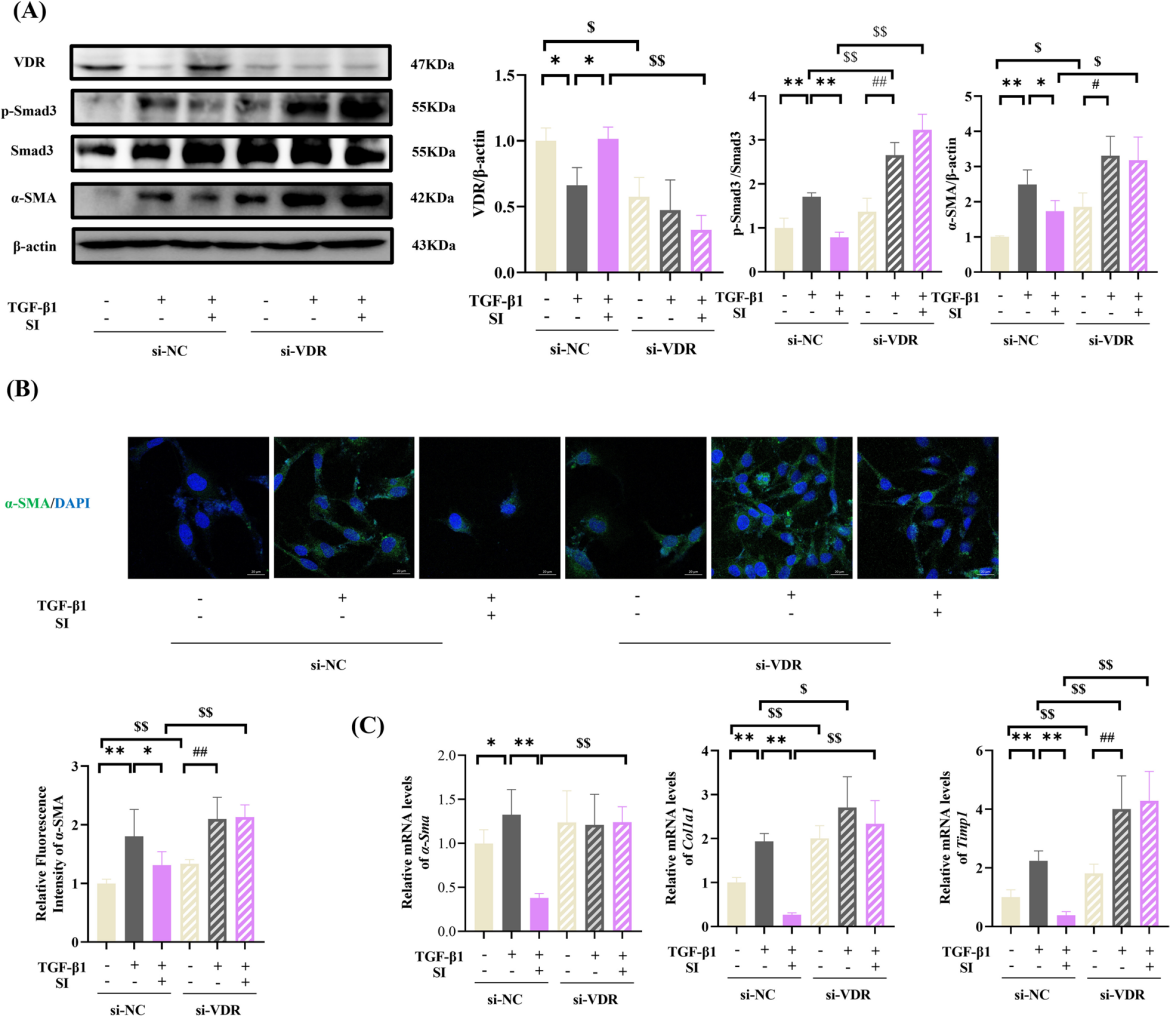
VDR knockout abolished the SI-induced inhibition of HSCs activation. **A** Western blot analysis (n = 3). **B** Immunofluorescence analysis of α-SMA. scale bar = 20 μm. (n = 6). **C** mRNA levels of fibrogenic markers (*α-Sma*, *Col1a1*, and *Timp1*). (n = 6) *P < 0.05, **P < 0.01 compared with si-NC + TGF-β1; $*P* < 0.05, $$*P* < 0.01 compared with si-NC; #*P* < 0.05, ##*P* < 0.01 compared with si-VDR + TGF-β1

Given the established role of the VDR in fatty acid metabolism regulation [48], we investigated the involvement of the VDR in the SI-mediated attenuation of hepatic fibrosis. The efficiency of siRNA-mediated VDR knockdown was confirmed in HSCs (Fig. 7A). SI treatment significantly upregulated VDR expression but suppressed Smad3 phosphorylation and α-SMA in TGF-β1-stimulated HSCs compared to TGF-β1-treated si-NC cells. Notably, VDR knockdown abolished SI's inhibitory effects on both Smad3 phosphorylation and α-SMA expression in TGF-β1-activated HSCs (Fig. 7B).

Compared with those in the si-NC control cells, the mRNA levels of *α-SMA*, *Col1 A1*, and *Timp1* in the si-VDR cells were increased (Fig. 7C). Importantly, SI failed to suppress TGF-β1-induced upregulation of these fibrogenic markers in VDR-deficient cells. These findings demonstrate that VDR activation is essential for the antifibrotic effects of SI, potentially through the modulation of Smad3 signaling and extracellular matrix regulation.

### VDR silencing abrogates SI-mediated lipid metabolic reprogramming in HSCs

**Fig. 8:**
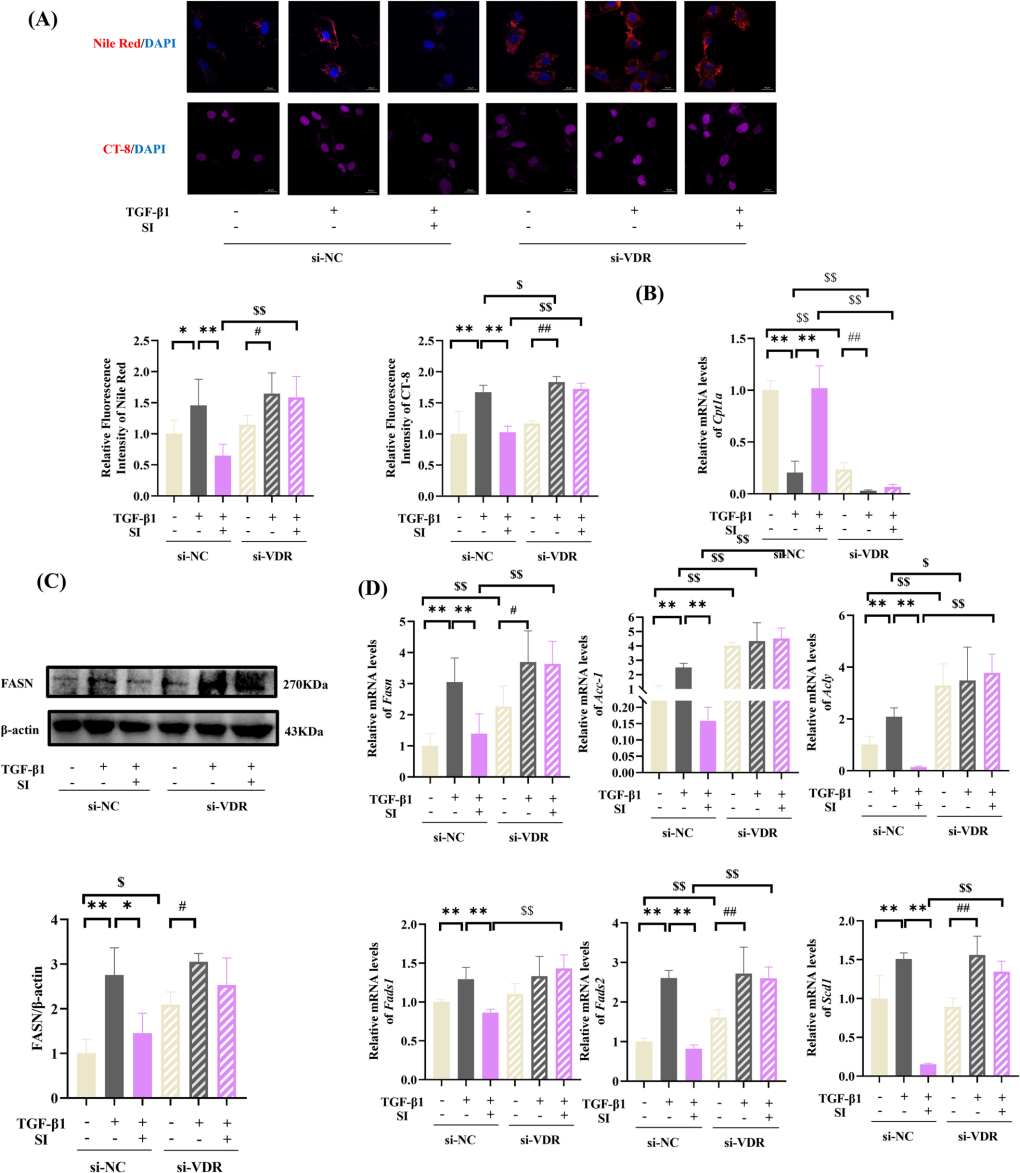
SI reprogramed fatty acid metabolism through activating VDR in TGFβ1-induced HSCs. **A** Lipid droplet accumulation. scale bar = 20 μm. **B** *Cpt1a* expression. **C** Western blot analysis of FASN. (n = 3). **D** mRNA levels of lipogenic enzymes (*Fasn*, *Acc-1*, and *Acly*) and fatty acid desaturases (*Fads1/2* and *Scd1*). (n = 6) *P < 0.05, **P < 0.01. *P < 0.05, **P < 0.01 compared with si-NC + TGF-β1; $*P* < 0.05, $$*P* < 0.01 compared with si-NC; #*P* < 0.05, ##*P* < 0.01 compared with si-VDR + TGF-β1

To investigate the role of VDR in SI-induced lipid metabolic reprogramming, we quantified Nile Red and CT-8 fluorescence quantification and detected increased lipid deposition in si-VDR-treated HSCs compared with si-NC controls (Fig. 8A). TGF-β1 cotreatment exacerbated lipid accumulation in si-VDR-treated cells, but SI failed to mitigate this effect. Molecular analysis revealed significant downregulation of the β-oxidation marker *Cpt1a* (Fig. 8B) and upregulation of lipogenic regulators (*Fasn*, *Fads1 Fads2*, *Acc-1*, *Acly*, and *Scd1*) in VDR-deficient cells (Fig. 8C, D). Notably, TGF-β1 stimulation elevated the expression of lipogenic regulators in si-VDR HSCs independent of SI intervention.

## Discussion

HSC activation drives liver fibrosis via fatty acid metabolic reprogramming, with VDR identified as a key transcriptional regulator. This study demonstrated that VDR deletion exacerbated fibrosis, increased TGF-β1/Smad3 signaling and disrupted lipid homeostasis by upregulating lipogenic/desaturase enzymes while suppressing the β-oxidation gatekeeper. Pathological VDR downregulation in TGF-β1-activated HSCs and fibrotic tissues highlights disease-linked regulation. Calcitriol-activated VDR normalized metabolic enzyme expression, reduced lipid/collagen accumulation, and inhibited Smad3 phosphorylation. Senkyunolide I, a novel VDR agonist, rebalances metabolism by suppressing lipogenesis/desaturation and enhancing CPT1 A, and its anti-fibrotic effects strictly VDR-dependent. These findings validate VDR as a therapeutic target and SI as a dual-action anti-fibrotic agent via VDR signaling.

The activation of HSCs serves as the principal cellular mechanism driving liver fibrogenesis [24]. Notably, CCl_4_ induction primarily exerts fibrogenic effects on the liver without causing fibrosis in other organs such as the kidney. Utilizing a CCl_4_-induced murine fibrosis model, we demonstrate that CCl_4_ administration triggers significant lipidomic remodeling through upregulation of lipogenic enzymes, establishing a pro-fibrogenic lipid microenvironment that amplifies HSC activation [6, 19]. This process concurrently activates the TGF-β1/Smad3 signaling axis, creating a self-reinforcing feed-forward circuit between fibrogenic signaling and metabolic dysregulation. Our findings corroborate previous reports of CCl_4_-induced Smad3 phosphorylation in hepatic tissue and TGF-β1-mediated HSCs activation [33]. Pharmacological restoration of fatty acid metabolism enzymes effectively attenuated collagen deposition, suggesting potential therapeutic mechanisms through metabolic modulation [27]. The mechanistic interplay between lipid overload and TGF-β1/Smad3 activation^26^, ^50^ may involve cholesterol-facilitated TGF-β receptor complex assembly [20] and saturated fatty acid-induced SMAD3 phosphorylation [16]. Further investigation is required to elucidate causal relationships between lipid metabolic dysregulation and fibrogenesis. Our findings position metabolic regulation as a novel therapeutic axis capable of disrupting the pathological cycle of HSCs activation and fibrotic matrix production.

Downregulation of VDR represents a key pathological feature in chronic liver diseases [14]. Studies demonstrate that VDR deficiency promotes spontaneous chronic liver inflammation and hepatic steatosis, whereas VDR activation mitigates fatty acid and cholesterol accumulation in obesity-associated models [1]. Mechanistically, ligand-activated VDR suppresses HSCs activation by modulating ECM production [15, 29]. Our findings further reveal that VDR depletion enhances the transcriptional activity of lipid metabolic enzymes, driving lipid accumulation that subsequently triggers TGF-β1 secretion and Smad3 phosphorylation. This aligns with prior studies demonstrating that vitamin D derivatives ameliorate hepatic fibrosis by inhibiting TGF-β/Smad signaling and HSC activation [7, 39]. Notably, VDR deficiency disrupts lipid homeostasis, elevating TC levels and promoting lipid deposition. Collectively, these data position VDR as a central regulator of lipid metabolism disorders that drive liver fibrosis progression.

The current clinical management of liver fibrosis primarily relies on antiviral nucleoside analogs combined with interferons, supplemented by anti-inflammatory/antioxidant agents like silymarin, ursodeoxycholic acid, and bicyclol [56]. Although vitamin D acts as a natural VDR agonist, its therapeutic application is constrained by hypercalcemia risks [45]. Rhizoma Chuanxiong (Ligusticum chuanxiong Hort.), a classical traditional Chinese medicine, has been documented for liver fibrosis treatment [41, 47]. Previous studies report that tetramethylpyrazine from Rhizoma Chuanxiong inhibits post-myocardial infarction cardiac fibrosis [53, 55]. Herein, SI, a principal bioactive component of Rhizoma Chuanxiong, exhibits potent anti-fibrotic efficacy in preclinical models. Through in vivo and in vitro fibrosis models, we demonstrate that SI inhibits HSCs activation and reduces ECM deposition. Mechanistically, SI selectively activates VDR to initiate fatty acid metabolic reprogramming, effectively mitigating lipid accumulation in HSCs and reversing fibrogenic activation via suppression of TGF-β/Smad3 signaling. Crucially, SI achieves VDR agonism without elevating serum calcium levels. This work elucidates the role of SI in modulating metabolic reprogramming and identifies this bioactive compound as a calcium-tolerant therapeutic candidate for liver fibrosis treatment.

## Conclusion

This study elucidates the critical role of VDR in hepatic fibrogenesis, particularly its novel mechanism of attenuating liver fibrosis through modulation of fatty acid metabolism in HSCs. Our findings demonstrate that VDR deficiency induces dysregulation of fatty acid metabolism, thereby activating the TGF-β/Smad signaling pathway to promote HSCs activation and excessive collagen deposition. Notably, SI intervention effectively reverses fibrotic progression by targeting VDR-mediated suppression of TGF-β signaling. This research not only reveals a previously unrecognized metabolic regulatory mechanism of VDR in liver fibrosis but also offers novel therapeutic avenues for combating hepatic fibrosis through metabolic reprogramming strategies.

## Supplementary Information

Additional file1

Additional file2

Additional file3

## Data Availability

Data will be made available on request.

## Abbreviations

HSCs: RHuman hepatic stellate cells
α-SMA: α-smooth muscle actin
COL1A1: Collagen type I alpha 1
TIMP1: Tissue inhibitor of metalloproteinase 1
ALP: Alkaline phosphatase
AST: Aspartate aminotransferase
ALT: Alanine aminotransferase
TBIL: Total bilirubin
ACC1: Acetyl-CoA carboxylase 1
ACLY: ATP-citrate lyase
FADS1/2: Fatty acid desaturase
FASN: Fatty acid synthase
SCD1: Stearoyl-CoA desaturase 1
CPT1A: Carnitine palmitoyltransferase 1A
TGF-β1: Transforming growth factor-β1
p-Smad3: Phosphorylated Smad3
ECM: Excessive extracellular matrix
SI: Senkyunolide I
SLB: Silybin
TVB: TVB3664
VD: Vitamin D
VDR: Vitamin D receptor
CCl4: Carbon tetrachloride
CETSA: Cellular thermal shift assay
HE: Hematoxylin – eosin
DMEM: Dulbecco’s modified eagle medium
FBS: Fetal bovine serum
WT: Wild-type
i.p.: Intraperitoneal
VDR-/-: VDR-knockout
qRT-PCR: Quantitative reverse transcription polymerase chain reaction
SD: Standard deviation
