# Supplementary figures and images for: Senkyunolide I suppresses hepatic stellate cell activation and liver fibrosis by reprogramming VDR-dependent fatty acid metabolism
Source: Chin Med. 2025 Jun 13;20:85. doi: 10.1186/s13020-025-01133-x (PMC12164082; doi:10.1186/s13020-025-01133-x)

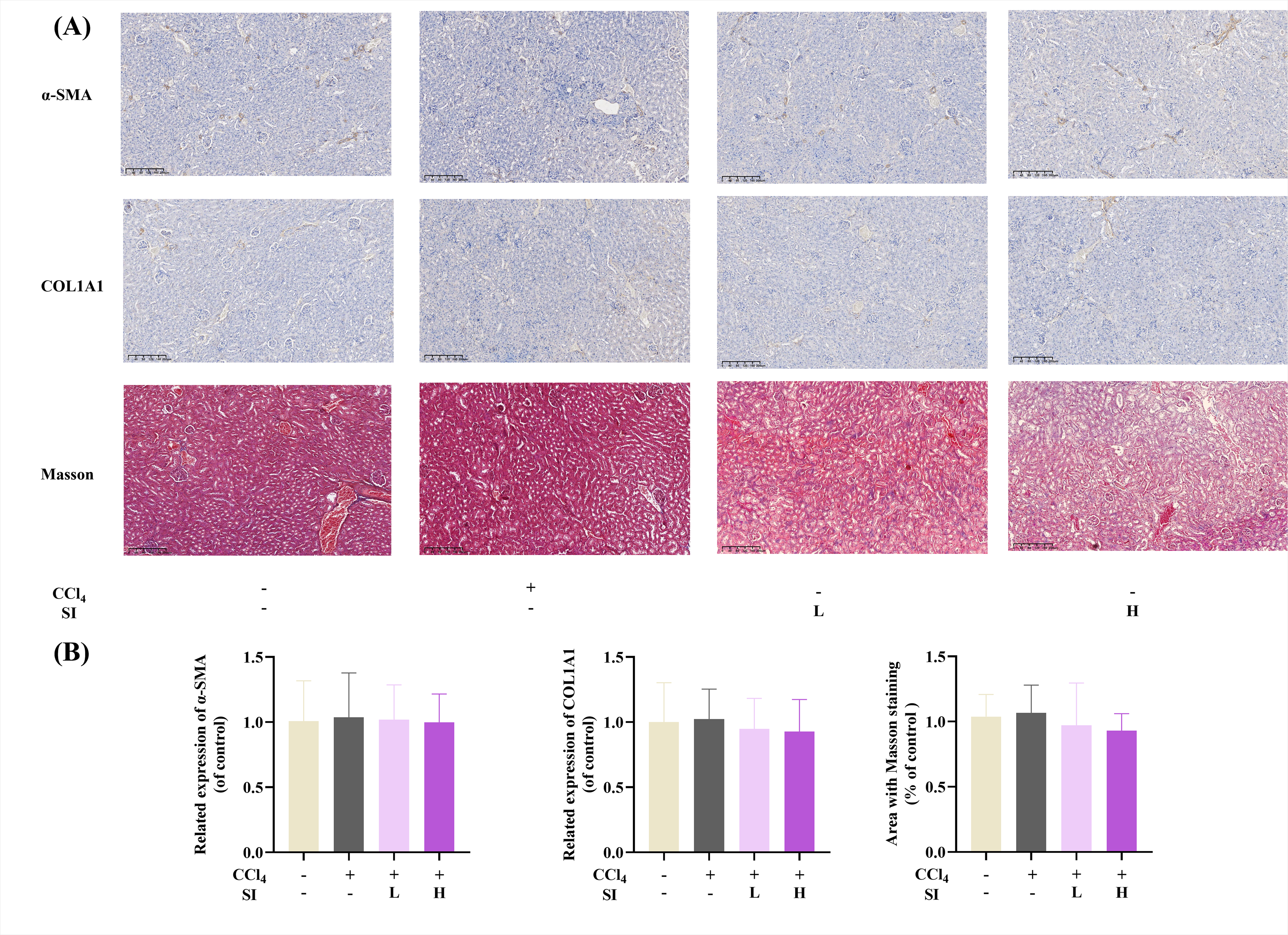

Supplement: Supplementary file 1 — Additional file1 [file 13020_2025_1133_MOESM1_ESM.tif]
