# Supplementary material for: Senkyunolide I suppresses hepatic stellate cell activation and liver fibrosis by reprogramming VDR-dependent fatty acid metabolism
Source: Chin Med. 2025 Jun 13;20:85. doi: 10.1186/s13020-025-01133-x (PMC12164082; doi:10.1186/s13020-025-01133-x)
Supplement: Supplementary file 2 — Additional file2 [file 13020_2025_1133_MOESM2_ESM.docx]

Table S1. Primer sequence

| Names | Sequence (5’-3’) |
| --- | --- |
| Human-*α-Sma* | F: CAGATGAAAGTGAGAACTCCAGGTC  R: GGCAGCCAGCATGATAGTAAGTG |
| Human-*Scd1* | F: AACCTGGCTTGCTGATGATGTG  R: AGGAGTGGTGGTAGTTGTGGAAG |
| Human-*Fads2* | F: CTGAGGAAGACGGCTGAGGAC  R: GTGCTGGAAGTGGCGATGATTC |
| Human-*Fads1* | F: CGTCCGCTTCTTCCTCACTTATG  R: TGAAGGCAGACTTGTGGACATTG |
| Human-*Acly* | F: AGCGAGCAGCAGACCTATGAC  R: CTGGCGTTGAGGAGGAAGTTTG |
| Human-*Acc-1* | F: CGTAGACACTGCCAGAACACTATTC  R: TAGGTGTGTGATTGTGCCATTTCC |
| Human-*Timp1* | F: GACCACCTTATACCAGCGTTATGAG  R: GGTCCGTCCACAAGCAATGAG |
| Human-*Col1a1* | F: AGGGCGACAGAGGCATAAAGG  R: ACAGGACCAGCATCACCAGTG |
| Human-*Fasn* | F: TTCTACGGCTCCACGCTCTTC  R: CTCAGGCTTGTCCTCCTCCAG |
| Human-*Cpt1a* | F: AGGCGACATCAATCCGAACATTCC  R: CGTCGTTTGCCAGAAGATTTGCG |
| Human-*Gapdh* | F: ACACCCACTCCTCCACCTTTG  R: TCCACCACCCTGTTGCTGTAG |
| Mouse-*α-Sma* | F: ATGACCCAGATTATGTTTGAGACCTTC  R: TCTCCAGAGTCCAGCACA |
| Mouse-*Scd1* | F: AGCCTGTTCGTTAGCACCTTCTTG  R: GCACCCAGGGAAACCAGGATATTC |
| Mouse-*Fads2* | F: TTCAGCGGGCACCTCAATTTCC  R: ACGGCTTCTCCTGGTATTCAATGC |
| Mouse-*Fads1* | F: TCCTGGTCTACCTGCTTCACATCC  R: CAACCTGCCTGAGCCTGAACTG |
| Mouse-*Acly* | F: TGTGATGAGCGAGGGCAGGAG  R: CTGGCAGGAATACTTGGGCAACC |
| Mouse-*Acc-1* | F: CCCAGAGATGTTTCGGCAGTCAC  R: GTCAGGATGTCGGAAGGCAAAGG |
| Mouse-*Timp1* | F: AAGGATTCAAGGCTGTGGGAAATG  R: GAAACTCTTCACTGCGGTTCTGG |
| Mouse-*Col1a1* | F: TCAGAGGCGAAGGCAACAGTC  R: GCAGGCGGGAGGTCTTGG |
| Mouse-*Fasn* | F: TGCCCGAGTCAGAGAACCTACAG  R: TCCATAGAGCCCAGCCTTCATC |
| Mouse-*Cpt1a* | F: AGGCGACATCAATCCGAACATTCC  R: CGTCGTTTGCCAGAAGATTTGCG |
| Mouse-*Gapdh* | F: AACTCCCACTCTTCCACCTTCG  R: TCCACCACCCTGTTGCTGTAG |
