# Supplementary material for: Senkyunolide I suppresses hepatic stellate cell activation and liver fibrosis by reprogramming VDR-dependent fatty acid metabolism
Source: Chin Med. 2025 Jun 13;20:85. doi: 10.1186/s13020-025-01133-x (PMC12164082; doi:10.1186/s13020-025-01133-x)
Supplement: Supplementary file 3 — Additional file3 [file 13020_2025_1133_MOESM3_ESM.docx]

Table S2. siRNA sequences

| Names | Sequence (5’-3’) |
| --- | --- |
| *si-NC* | F: UUCUCCGAACGUGUCACGUTT  R: ACGUGACACGUUCGGAGAATT |
| *si-VDR* | F: GGACUGAAGAAGCUGAACUTT  R: AGUUCAGCUUCUUCAGUCCTT |
